# Evaluation of the socioprofessional consequences of thoracic outlet syndrome

**DOI:** 10.1186/s13104-023-06448-2

**Published:** 2023-09-11

**Authors:** Charlotte Logiou, Xavier Demondion, Vincent Tiffreau, Valérie Wieczorek, André Thevenon

**Affiliations:** 1 Pole RRSS, CHU Lille, Lille, F-59000 France; 2 Anatomy Laboratory, University of Lille, Lille, F-59000 France; 3 Radiology and Musculoskeletal Imaging Division, CHU Lille, Lille, F-59000 France; 4 EA 7369URePPS, University of Lille, Lille, F-59000 France

**Keywords:** Thoracic outlet syndrome, Work, Professional, Impact

## Abstract

**Purpose:**

Thoracic outlet syndrome (TOS) is a ductal syndrome that can have a significant functional impact. Various studies have highlighted positional factors and repetitive movements as risk factors for the development of TOS. However, there are few literature data on the socioprofessional consequences of TOS.

**Methods:**

We performed a prospective, cross-sectional, descriptive, multicentre study of workers having received a Doppler ultrasound diagnosis of TOS between December 17th, 2018, and March 16th, 2021. Immediately after their diagnosis, patients completed a self-questionnaire on the impact of TOS on their work activities. We assessed the frequency of TOS-related difficulties at work and the associated socioprofessional consequences. Trial Registration Number (TRN) is NCT03780647 and date of registration December 18, 2018.

**Results:**

Eighty-two participants (95.3%) reported difficulties at work. Seventy-seven of the participants with difficulties (94%) worked in the tertiary sector; these difficulties were due to prolonged maintenance of a posture, carrying loads, and repetitive movements. Although the majority of participants experienced organizational problems and lacked support at work, few of them had approached support organizations, expert and/or healthcare professionals.

**Conclusions:**

TOS was almost always associated with difficulties at work (95.3%). However, poor awareness of sources of help or a perceived lack of need may discourage people with TOS from taking steps to resolve these difficulties. It is clear that the socioprofessional management of TOS requires significant improvements.

**Supplementary Information:**

The online version contains supplementary material available at 10.1186/s13104-023-06448-2.

## Introduction

Thoracic outlet syndrome (TOS) is a ductal syndrome in which compression of the brachial plexus, artery and/or subclavian vein can be responsible for neurological, arterial, and/or venous clinical signs. In the majority of cases, TOS has a predominant neurogenic component [1, 2]. Thoracic outlet syndrome is more common in women (female/male sex ratio: 4:1) and mainly develops between the ages of 20 and 40 [3, 4].

The pathophysiology, diagnosis and treatment of TOS are subject to much debate [3, 5]. The condition’s pathophysiology is multifactorial and poorly characterized. Postural disorders and movement-related factors that cause hypertrophy/spasms of the shoulder girdle muscles appear to contribute to the disease [6–8]. These factors can be work-related, due to the prolonged maintenance of poor posture or repetitive movements with the upper limb in abduction, externally rotated, or held above the shoulder level. Carrying a heavy load on the shoulder (and thus lowering it) has also been incriminated [9–11].

Most studies of work-related problems associated with TOS were retrospective or cohort-based and have not provided enough evidence of a causal etiological role of occupational factors; hence, TOS is not recognized (in France, at least) as an occupational illness [9, 12, 13].

Furthermore, a number of studies have assessed difficulties at work caused by the TOS (with a focus on occupational incapacity and the amount of time off work) but did not analyze more precisely the nature and degree of inconvenience caused [14, 15].

It is important to study TOS-related difficulties at work, in order to implement appropriate preventive measures and to raise awareness of this potential occupational illness.

The primary objective of the present study was therefore to assess the frequency of difficulties at work encountered by patients with TOS. The secondary objectives were to identify the socioprofessional consequences of these difficulties and the approaches and solutions implemented in response. Lastly, we assessed signs and symptoms of TOS and the type of work activities involved.

## Material and method

This was a prospective, non-interventional, observational, multicentre study.

Trial Registration Number (TRN) is NCT03780647 and date of registration December 18, 2018.

### Population

The study population consisted of adult patients (aged 18 or over) having received a Doppler ultrasound diagnosis of TOS in three radiology or angiology centres in the Hauts de France region of northern France (Lille University Hospital and Louvière Hospital in Lille, and a private angiology practice in Marcq-en-Baroeul) between December 17th, 2018, and March 16th, 2021. All the participants were in work at the time of the study or had been in work after the onset of their symptoms. Individuals reporting upper limb problems other than those ascribed to TOS and that impaired their work abilities were subsequently excluded.

### Conduct of the study

The patients had undergone a Doppler ultrasound assessment of the upper limbs for suspected TOS in one of the three radiology or angiology centres. The primary diagnostic criterion was arterial compression of at least 80% during sensitization manoeuvres (shoulder abduction and retropulsion).

Following the Doppler ultrasound diagnosis, the patient was invited to participate in the study. After the inclusion and non-inclusion criteria had been checked, the patient gave his/her written, informed consent. Immediately after the diagnosis, each of the participants completed a self-questionnaire on the socioprofessional consequences of TOS (supplemental data) on-site in the radiology or angiology clinic. The questionnaire took about 30 minutes to complete, it was specifically developed for this study.

### Statistical analysis

The statistical analyses were carried out by the methodology, biostatistics and data management unit at Lille University Hospital, using SAS software (version 9.4, SAS Institute Inc., Cary, NC, USA). Qualitative variables were described as the frequency (percentage). Quantitative variables were described as the mean ± standard deviation (if normally distributed), the median [interquartile range (IQR)], or the mean (range).

Using Fisher’s test; we compared the work sector data for our study population with those for the French general population in employment in 2017 [16].

The required sample size was initially estimated to be 120. At the time when the study protocol was drafted, we expected that 20 to 30% of the TOS patients would experience difficulties at work. We estimated that a study population of 120 would provide an accuracy of 7.5%. However, given the much higher proportion of affected patients and the recruitment difficulties caused by the concurrent COVID-19 health, the inclusion process was stopped after 100 people had been recruited.

## Results

Of the 100 patients included by the investigating centres, 14 were excluded because they had another upper limb pathology that impaired their work abilities. Seven of the 14 exclusions were linked to distal nerve compression.

### The study population

The study population was predominantly female (87%), and the mean age was 40. The median (range) time interval between symptom onset and diagnosis was 24 months (0; 282) (Table 1). The best-represented educational level was “high school diploma or up to two years of higher education” (38 (44.2%) of the participants), followed by “three or four years of higher education” (22 (25.6%) of the participants).

**Fig. 1:**
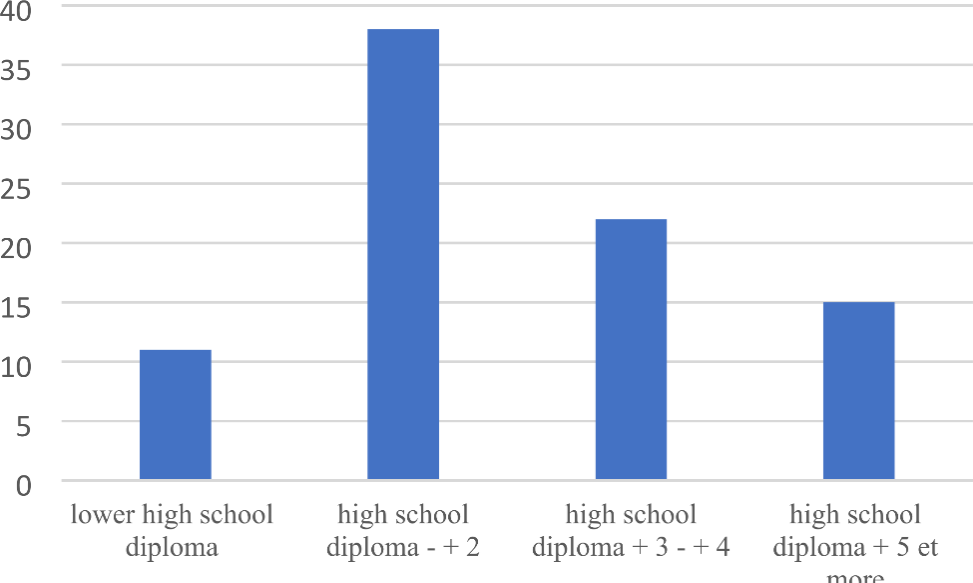
Distribution of educational grade levels in the study population (n = 86)

27 (31.4%) patients reported loss of income directly related to TOS. (Fig. 1)

**Table 1:** Characteristics of the study population

Patients (N)	86
**Sex**	
Female, n (%)	75 (87.2)
Male; n (%)	11 (12.8)
**Age (years)**	
mean ± standard deviation	39.9 ± 10.0
range	(18; 64)
**Time between onset of symptoms and diagnosis (months)**	
median [IQR]	24.0 [12.0; 60.0]
range	(0; 282 )

### Primary outcome

The percentage [95% confidence interval] of patients with work difficulties directly related to TOS was 95.35% [0.909; 0.998] (Table 2).

**Table 2:** Frequency and percentage of study participants with TOS-related difficulties at work

Difficulty at work	Number	Percentage	Cumulative frequency	Cumulative percentage
**No difficulty**	4	4.65	4	4.65
**difficulty**	82	95.35	86	100.00

### Secondary outcomes

#### Impact at work (table 3)

Forty-nine of the 86 patients (57%) had taken time off work because of TOS, for an average of 17 weeks. Forty-six patients (56%) had organizational difficulties at work; these were mainly due to treatment and follow-up of the disease (in 85% of cases), difficulties in enforcing the recommendations given to the employer by an occupational physician, and difficulties moving to part-time work. Fifty-three patients (65%) felt misunderstood and/or not supported in their work environment, and among these patients 15,9% even felt harassed by their hierarchy. Fourteen patients (17%) reported loss of employment or a non-renewal of a contract in relation to TOS; this was mainly due to resignation, redundancy or mutually agreed termination of employment (in 8 of the 14 cases), dismissal for medical incapacity (in 5 cases) or non-renewal of an employment contract (in 3 cases). Twenty-nine patients (35%) also described other difficulties; a general deterioration in working conditions (in 10% of cases), disabling fatigue (8.5%), obstacles to professional development (promotions, redeployment, training, etc.), and anxiety-depressive disorders.

**Table 3:** The impact of TOS at work in the study population (n = 82)

Patients (N)	82
Time off work (weeks)	
mean ± standard deviation	10.1 ± 15.5
range	[0; 72]
Organizational difficulties at work, n (%)	46 (56.1%)
Harassment/lack of support, n (%)	53 (64.6%)
Loss of employment or contract non-renewal, n (%)	14 (17.1%)
Other occupational difficulties, n (%)	29 (35.4%)

### Initial disabling symptoms (Fig. 2)

**Fig. 2:**
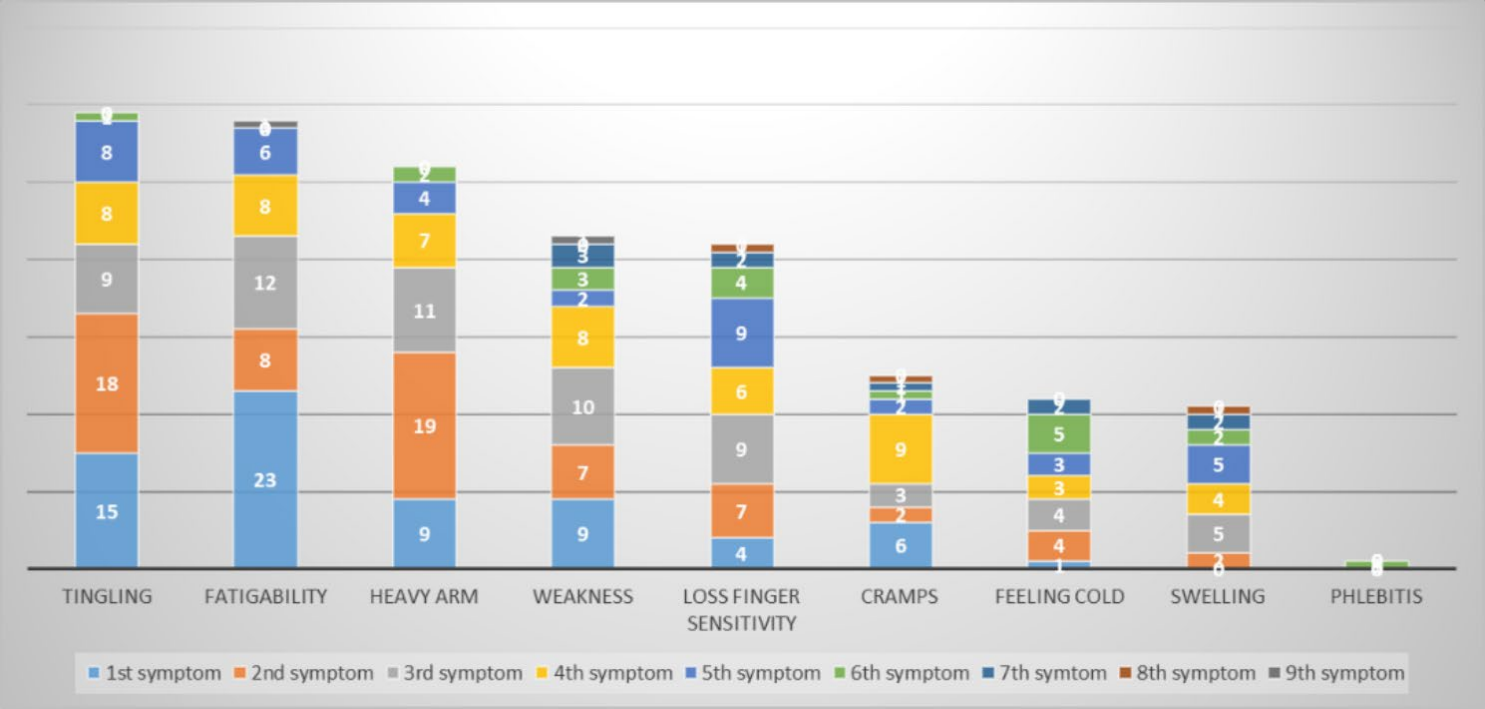
Symptoms at TOS onset among the study participants with difficulties at work (n = 82)

The most common initial symptoms were tingling in the hands (in 88% of patients), fatigability with repetitive movements (in 84%), a feeling of heaviness in the arm (in 79%), constant weakness (in 65%), and loss of sensitivity in the fingers (in 63%) (Fig. 2). The most frequently reported main disabling symptoms were fatigability with repetitive movements (in 30.5% of the participants) and tingling in the hands (in 25.5%).

### Type of work activities at symptom onset

**Fig. 3:**
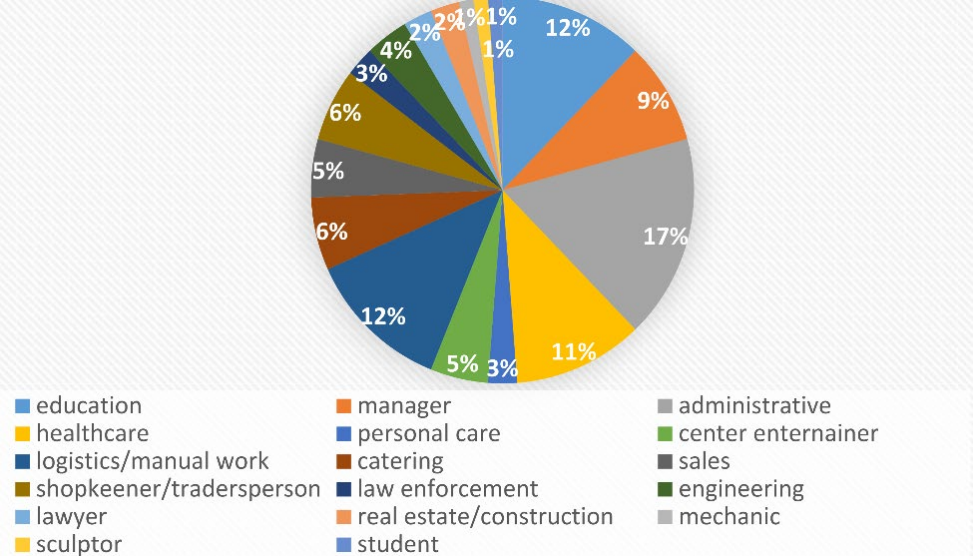
Work activity at symptom onset among study participants with TOS-related difficulties at work (n = 82)

When considering participants who experienced work difficulties at the onset of TOS, the most frequent types of work activity were the administrative sector (n = 14 (17%)), the education sector (n = 10 (12.2%)), logistics and manual work (n = 10 (12.2%)), and the health sector (n = 9 (11%)). (Fig. 3). Seven of the participants were managers or executives in various sectors.

We compared the work sector data for our study population with those for the general French population in employment in 2017 (16). The only significant differences concerned the industrial sector; the proportion of workers in the industrial sector was significantly higher (by 12.6% points; p < 0.001 in the French general population than in our study population.

### Tasks at work

**Fig. 4:**
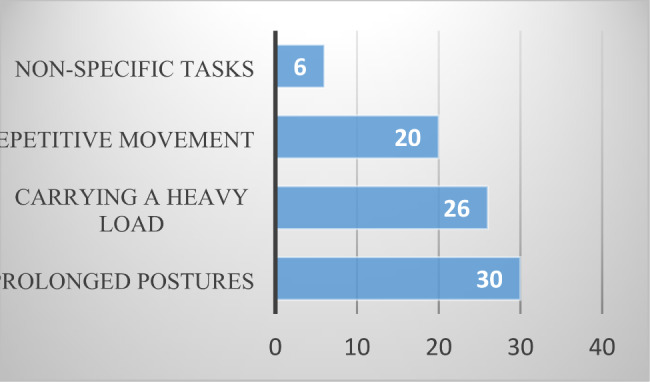
The most disabling tasks at work

The most disabling and symptom-triggering tasks were prolonged maintenance of a posture (in 30 (37%) of cases, including working on a computer), sitting at a desk (4 patients), carrying a heavy load on the shoulder, above shoulder height or at arm’s length (in 26 (32%) of cases), repetitive movements (in 20 (24%) of cases, including precise gripping tasks for 8 patients, elevation, abduction and retropulsion of the shoulder (such as writing on a board) in 8 patients, and all work-related tasks (6 patients (7%)) (Fig. 4).

### Approaches and solutions for addressing difficulties at work

Twenty-seven patients (33%) had consulted an occupational physician, who had recommendation adaptation of the work activities in 72% of cases (Table 4). Of the 55 patients (67%) who did not consult an occupational physician, 29 patients (53%) stated that they did not know that he/she might be of assistance, whereas 10 patients (18%) did not feel the need. 8 patients (14.5%) thought that they did not have an occupational physician or were afraid of losing their job (7 patients (12. 7%)). One patient did not trust the occupational physician.

Fifty (61%) of the patients consulted their general practitioner or a specialist physician: 28 (56%) received a doctor’s note for a short period of time off work, 15 (30%) received a note for a long period of time off work (more than 3 months), and 15 (30%) also requested a change in their treatment or dose.

Sixteen (19.5%) of the patients contacted their line managers with a view to changing their work activities, requesting a part-time contract, changing jobs, or mutually agreed termination of employment. Eleven (13%) of patients contacted the county health services in order to be classified as a handicapped worker. Five (6%) contacted the state social security body and requested chronic disease status, disability allowance, occupational disease status, or an appointment with a social worker.

**Table 4:** The procedures implemented by patients impacted at work by TOS

Patients (N)		82
Consultation with an occupational physician, n (%)		27 (32.9%)
Consultation with a general practitioner or a specialist physician, n (%)		50 (61%)
	Short-or medium-term sick leave, n (%)	28 (56%)*
	Long-term sick leave, n (%)	15 (30%)*
	Therapeutic adaptation, n (%)	15 (30%)*
Requests made to line managers, n (%)		16 (19.5%)
	Job adaptation, n	8
	Part-time work, n	6
	substation loading, n	3
	Conventional termination, n	1
Requests made to the county health services, n (%)		11 (13.4%)
	Application for handicapped worker status, n	11
	Application for a disability allowance, n	0
	Other applications, n	0
Requests made to the social services and the state health insurance body, n (%)		5 (6.1%)
	Invalidity status, n	1
	Reassessment of disability category, n	0
	Appointment with a social worker, n	1
	Occupational illness, n	1
	Chronic disease status, n	2
Other/informal solutions, n (%)		28 (34.1%)

* as a percentage of the patients having consulted a general practitioner or a specialist

### Situations in which a solution was not found (table 5)

61 of the 82 patients (74%) had not found a solution for their difficulties at work. 56% of these patients found the situation bearable, while 39% had psychological difficulties: some had even left or lost their jobs or experienced financial and/or personal difficulties.

Of these 61 patients, 24 (39%) did not feel the need to make approaches, 21 (34%) were not aware that the above-mentioned organizations and healthcare professionals could be of assistance, and others did not look for a solution for other reasons (fear, discouragement, or fatigue). 9 patients (14.7%) considered that they had not been helped (or not helped enough), despite having initiated actions.

**Table 5:** Reasons for the lack of solution for patients on whom TOS had a socioprofessional impact

Patients (N)		82
No solution found, including:		61 (74.4%)
	loss of employment, n (%)	9 (14.7%)*
	bearable situation, n (%)	34 (55.7%)*
	psychological difficulties, n (%)	24 (39.3%)*
	Separation, n (%)	2 (3.3%)*
	financial loss, n (%)	2 (3.3%)*
	failure to retrain, n (%)	2 (3.3%)*
Reasons for the lack of solution		
	not being aware of the available help, n (%)	21 (34.4%)*
	did not feel the need, n (%)	24 (39.3%)*
	other reasons, n (%)	7 (11.5%)*
	the organizations did not provide any help, n (%)	3 (4.9%)*
	the organizations did not provide sufficient help, n (%)	6 (9.8%)*

* as a percentage of the 61 patients who had not found a solution

## Discussion

We found that over 95% of our patients with TOS experienced difficulties at work. Despite this situation, very few had contacted support organizations, experts and/or healthcare professionals with a view to solving these difficulties.

When considering demographic characteristics, the study population was quite similar to those described in the literature on TOS: clear female predominance (male:female ratio: 1:8, vs. 1:4 in the literature) [3, 17], a mean age of 40 [12, 17]. The time interval between the first symptoms and diagnosis (2 years) was particularly long. This diagnostic delay has already been highlighted in the literature and is mainly due to serial misdiagnosis [1, 12]. The symptoms considered to be the most disabling at work were tingling in the hand and fatigability upon repetitive movement, as commonly reported in the literature [1, 18].

We found that a high proportion of our patients’ difficulties at work were directly related to TOS. To the best of our knowledge, this is a new finding. These difficulties were probably due to the presence of occupational factors leading to muscle strain and postural disorders, which in turn can trigger the symptoms of TOS. Hence, it is unsurprising that the patients faced difficulties at work [7, 8].

Given the long diagnostic delay, TOS had a major impact in terms of difficulties at work and the lack of support in the work environment. The deterioration of working conditions led to repeated leaves of absence and obstacles to professional development - including even loss of employment and/or income.

It is difficult to compare our present results with the literature data. In contrast to previous studies, our survey was carried out at the time of diagnosis. Furthermore, we used different criteria.

In our study, more than half of the respondents had taken time off work. The average time off work was 10.2 weeks; this is much lower than the literature values. For example, the average time off work was 10.7 months in a study of 46 patients [15]. This disparity might be due to methodological differences; the patients in our study had just been diagnosed, whereas the patients in the other studies were attending rehabilitation centres or were due to undergo surgery – suggesting that the TOS in the latter cases was long-standing and particularly disabling.

The difficulties reported to us might be due to a lack of consideration and a lack of acknowledgement of the patients’ condition by healthcare professionals (with a long diagnostic delay and inadequate care) and in the work environment. Despite the difficulties expressed here, few of the patients had contacted social and occupational services. Only a third of the patients had contacted an occupational physician, and only 20% had contacted their line managers (mainly with a view to changing their work activities). Similarly, few patients had contacted support organizations, such as the county health services and the state social security body. This situation was also highlighted in a study of 149 patients with work-related musculoskeletal disorders (MSDs): only 32.9% had consulted an occupational physician at start of their illness and had been referred to a specialist occupational medicine centre [19].

Furthermore, a high proportion of our patients with difficulties at work had not found a solution, and the repercussions were quite varied. More than half of the patients found the situation to be bearable, while others experienced emotional problems. Several studies have found that psychosocial factors are predictive of MSDs as well as being risk factors for workplace accidents and absenteeism [20, 21]. The emotional difficulties reported in the present study might be risk factors for (or consequences of) TOS, as has been described for MSDs.

There were two main explanation for the patients’ lack of initiatives. Some of the patients did not feel that they needed because they were not greatly impacted. Others lacked information and were not aware of the potentially available help and the procedures that could be implemented.

With regard to the types of work activity concerned, 70% of our patients performed sedentary work and 30% performed physical work. 41% of the patients had intellectual and scientific jobs (nurses, teachers, managers, etc.) and 29% had administrative jobs (secretaries, accountants, administrative assistants, etc.). Similar proportions (i.e. a majority of people with intellectual and scientific jobs) have been reported by Lindgren [17] and Thevenon and al [14] but not by Nael [15]. However, methodological differences mean that it is difficult to compare studies in the workplace: the time since symptom onset and the occupational classifications differed from one study to another.

Our survey respondents reported much the same occupational risk factors as in the literature: the prolonged maintenance of a posture, carrying heavy loads (either on the shoulder or at arm’s length), repetitive movement (either closely controlled gripping tasks or shoulder elevation/abduction/retropulsion) [9, 11, 22]. Although the prolonged maintenance of a posture was mostly due to computer use, few studies have identified this as a risk factor for TOS. However, Reinstein [23] stated that computer use predisposes people to postural disorders and muscle imbalance at the neck/shoulders, which would promote the development of TOS.

Occupations with these risk factors therefore appear to be more prone to TOS, although the association between TOS pathogenesis and occupational exposure has yet to be demonstrated. This prompts the question of whether occupational exposure is a risk factor for TOS pathogenesis or merely triggers symptoms that are already present.

The study also had notable strengths. Firstly, we addressed a theme for which literature data are scarce - especially with regard to newly diagnosed patients. Secondly, we performed a robust, prospective, multicentre study, with a large population and a comprehensive questionnaire.

In order to gain a better understanding of the association between TOS and difficulties at work, it would be interesting to compare patients suffering from TOS with a control group.

### Limitations

Our study had some limitations. Firstly, study participants were included after a Doppler ultrasound diagnosis of TOS; this might have introduced selection bias and a lack of representativity. Secondly, the questionnaire was filled out on a voluntary basis and required a good level of French; this might have biased the replies concerning socioprofessional difficulties and the impact of TOS. Lastly, the data might have suffered from recall and information bias: half of the patients had been experiencing symptoms for more than 2 years, and experiencing difficulty at work is partly subjective.

## Conclusions

Although occupational risk factors for TOS have been studied for several years, there are few data on the socioprofessional difficulties experienced by these patients. The present study highlighted a high prevalence of difficulties at work (95%) among patients newly diagnosed with TOS. Despite these difficulties, few patients contacted support organizations, experts and/or healthcare professionals with a view to finding solutions.

Although this lack of contact was explained (for some patients) by the lack of a perceived need, many other patients lacked information on support and on the syndrome itself. It is clear that that the socioprofessional management of TOS requires significant improvements.

We identified computer use as a risk factor for TOS, however few studies had identified it. The risk factors at the time of diagnosis deserve further study.

### Electronic supplementary material

Below is the link to the electronic supplementary material.

Supplementary Material 1

## Data Availability

Data and materials are available by mail from Charlotte Logiou.
