# Supplementary material for: Evaluation of the socioprofessional consequences of thoracic outlet syndrome
Source: BMC Res Notes. 2023 Sep 11;16:207. doi: 10.1186/s13104-023-06448-2 (PMC10496342; doi:10.1186/s13104-023-06448-2)
Supplement: Supplementary file 1 — Supplementary Material 1 [file 13104_2023_6448_MOESM1_ESM.docx]

Non-Interventional Study No. 2017-26 entitled **" Evaluation of the socioprofessional consequences of thoracic outlet syndrome"**

**INCLUSION**

**PATIENT INITIALS: │__││__│ (first name) - │__││__│ (last name)**

**DATE OF BIRTH: │__│__│/│__│__│/│__│__│__│__│**

**PATIENT NUMBER: │__│__│- │__│__│__│**

**GENDER: ○ M ○ F**

**DATE OF INCLUSION: │__│__│/│__│__│/│__│__│**

**INCLUSION CENTER: │0 │__│**

**INCLUSION CRITERIA**

|  | |
| --- | --- |
| - **Patient over 18 years old.** | **○ YES ○ NO** |
| - **Patient employed or formerly employed** | **○ YES ○ NO** |
| - **Patient in whom a thoracic outlet syndrome has just been diagnosed on Doppler ultrasound: appearance of arterial compression of at least 80% during sensitization maneuvers (shoulder abduction and retropulsion) on Doppler ultrasound .** - **The patient has signed the non-objection form** - **Patient is willing to comply with all study procedures and duration.** | **○ YES ○ NO**  **○ YES ○ NO**  **○ YES ○ NO** |
| - **Patient affiliated with a social security scheme.** | **○ YES ○ NO** |

**NON-INCLUSION CRITERIA**

|  | |
| --- | --- |
| - **Patient with cognitive disorders, dementia, severe comprehension disorders linked to a phasic or psychic disorder).** | **○ YES ○ NO** |
|  |  |
| - **Illiterate patient** - **Patient with a significant impairment of visual abilities.** | **○ YES ○ NO**  **○ YES ○ NO** |

**EXCLUSION CRITERIA**

|  | |
| --- | --- |
| - **Person reporting other upper extremity problems affecting their professional abilities.** | **○ YES ○ NO** |
|  |  |
|  |  |

**PATIENT CHARACTERISTICS**

**DATE FILLED: / / INITIALS:**

**SEX: Male / Female AGE:**

**Date of onset of symptoms: ………………………………………**

**Date of diagnosis? …………………………………………………………**

**1) Do you currently have another diagnosed disease affecting your upper extremities that interferes your professional activity?**

**□ YES □ NO**

**If yes, what is the diagnosis? ................................**

**2) Were you employed at the time or since the appearance of the first signs of illness**

**□ YES □ NO**

**If yes: What was your job (as precisely as possible)**

**…………………………………………………………………………………………....**

**If you checked NO, the questionnaire is not adapted to your situation. We thank you for your motivation and participation.**

**If YES, please answer the following questions:**

**DIFFICULTIES ENCOUNTERED AT WORK DUE TO THORACIC OUTLET SYNDROME**

1) Generally speaking, has your income been affected by your illness?

□ NO, my income has not been affected overall by my illness

□ YES , my income has rather increased (career development, compensation through pensions, etc.)

□ YES, my income has rather decreased (job loss, decrease in working hours, etc.)

2) Initial qualification level:

☐ Level below Bac

☐ Bac to Bac +2 level

☐ Level Bac +3 +4

☐ Level Bac +5 and more

3) During your various jobs since the onset of the first symptoms of the disease, have you been in difficulty because of the disease or its symptoms?

□ NO --> ***if NO, thank you for your participation, the questionnaire is over.***

□ YES

4) Absenteeism: ……… ☐ months or ☐ weeks since the onset of the illness

5) □ Organizational difficulties at work.

5.1 □ Difficulties coordinating illness monitoring or care with work

5.2 □ Difficulties in enforcing job or time restrictions requested by the occupational physician

5.3 □ Difficulties in obtaining requested part-time work due to illness

6) □ Lack of understanding and/or support of the professional entourage toward the disease

6.1 □ Lack of support from colleagues

6.2 □ Lack of support from the hierarchy

6.3 □ Lack of support from the occupational physician

6.4 □ Harassment, derogatory remarks from colleagues related to illness

6.5 □ Harassment, disparaging remarks from superiors related to illness

6.6 □ Other situation of lack of understanding of the professional environment

7) □ Job loss or non-renewal of contract directly related to the illness

7.1 □ Loss of employment due to medical incapacity related to illness

7.2 □ Loss of employment by resignation, agreed termination or amicable termination due to illness

7.3 □ Non-renewal of a fixed-term contract or a temporary contract due to illness

7.4 □ Dismissal experienced as directly related to illness

7.5 □ Other reason for job loss directly related to illness

8) □ Other professional difficulty not mentioned (please specify concisely below, for example deterioration of working conditions, obstacle to professional promotion, etc.)

…………………………………………………………………………………………………………………………… ………………………………………

9) What are the symptoms directly related to your illness that are or were responsible for your difficulty(ies)? (several symptoms possible. If several answers: put a 1 in front of the most disabling symptom, a 2 in front of the next one, …. From the most disabling to the least disabling)

| Fatigue on repetition of movements |  | Heavy arm feeling |  | Tingling in the hands |  |
| --- | --- | --- | --- | --- | --- |
| Cramps in the upper limb(s) |  | hand swelling |  | Loss of finger sensitivity |  |
| Sensitivity to cold (white or blue hands) |  | Phlebitis of the upper limb |  | Weakness permed |  |

10)

10.1) What was your profession when you were confronted with the symptom that you classified as No. 1? (clearly and precisely) For example “college professor” rather than “teacher”

.................................................. .................................................. ........................

10.2) Which work task was mainly difficult?

.................................................. .................................................. ........................

11) Have you had recourse to the occupational/prevention physician for your difficulties?

□ YES □ NO

If NO, why ?

□ I didn't feel the need

□ I didn’t have an occupational physician (self-employed...)

□ I don't trust the occupational physician, I'm afraid he or she won't respect medical secrecy

□ I was afraid of losing my job

□ I didn't know that the occupational physician could help me

12) What solutions or steps have you taken to improve the situation or get out of your difficulties? (several answers possible including in each category)

12.1 □ Administrative procedures with the MDPH (Departmental House for Disabled Persons)

12. 1.1 □ RQTH request (recognition as a disabled worker)

12. 1.2 □ Application for disabled adult allowance

12. 1.3 □ Other disability compensation benefits

12.2 □ Approach to the treating physician or specialist

12. 2.1 □ Short or medium-term sick leave (less than 3 months)

12. 2.2 □ Long-term sick leave

12. 2.3 □ Treatment adaptation

12.3 □ Proceeding with the occupational physician or prevention physician

12. 3.1 □ Request for adaptation of position or schedule (on the initiative of the patient or the occupational physician)

12. 3.2 □ Request for reclassification (at the initiative of the patient or the occupational physician)

12. 3.3 □ Other request to the occupational physician specify ......................

12.4 □ Procedures in relation to your social protection organization (social security, etc.)

12.4.1 □ disabling

12.4.2 □ reassessment of disability category

12.4.3 □ Other procedures with social security (to be specified): ........................

12.5 □ Patient’s approaches to hierarchy

12. 5.1 □ Requesting a transfer or change of position

12. 5.2 □ Application for part-time work

12. 5.3 □ Other request to line management (please specify) ...............................

12.6 □ Personal solution or with help from colleagues (self-help, not formalized)

12.7 □ Other solution provided (to be specified) ......................................... ...................

12.8 □ I did not find a solution (multiple answers possible):

12. 8.1 □ I did not find a solution and I left/lost my job to escape the difficulties

12. 8.2 □ I did not find a solution but I found the situation bearable despite the difficulties

12. 8.3 □ I have not find a solution and I had moral difficulties (depression, anxiety….) because of this professional difficulty

12. 8.4 □ I did not find a solution and I had other problems because of my professional difficulties (specify problems).................. .................................

13 ) If you haven't found a solution yet, was it for one of the following reasons?

13.1 □ I did not know that there were structures that could provide me with help (occupational medicine, MDPH, social security, …)

13.2 □ I did not take any steps for another reason (fear, discouragement, fatigue...) but I knew of the existence of structures that could help me.

13.3 □ I didn't feel the need to take these steps.

13.4 □ The various structures/participants did not help with my difficulties (in this case, please specify the structure requested: MDPH, occupational medicine, social security, hierarchy….

13.5 □ The various structures I applied to provided the help I requested but it was not enough to keep my job or improve the situation.

14) Are you currently retired?

□ YES □ NO

If NO, are you currently employed?

□ YES □ NO

If YES, are you currently experiencing work-related difficulties with thoracic outlet syndrome?

□ YES, please specify which ones ( see question II.2):

| Fatigue on repetition of movements |  | Heavy arm feeling |  | Tingling in the hands |  |
| --- | --- | --- | --- | --- | --- |
| Cramps in the upper limb(s) |  | hand swelling |  | Loss of finger sensitivity |  |
| Sensitivity to cold (white or blue hands) |  | Limb phleb |  | Weakness permed |  |

**END OF STUDY**

Was the inclusion successfully completed:**🞏** Yes **🞏** No

**I certify that the data reported in this observation booklet is complete and accurate.**

**Name of the investigator: …………………………………………………………………**

**Date: |__|__|-|__|__|-|__|__|__|__|**

**Signature of the investigator:**
